# Emergency total thyroidectomy due to non traumatic disease. Experience of a surgical unit and literature review

**DOI:** 10.1186/1749-7922-7-9

**Published:** 2012-04-11

**Authors:** Mario Testini, Francesco Logoluso, Germana Lissidini, Angela Gurrado, Giuseppe Campobasso, Rocco Cortese, Giuseppe Massimiliano De Luca, Ilaria Fabiola Franco, Alessandro De Luca, Giuseppe Piccinni

**Affiliations:** 1 Department of Biomedical Sciences and Human Oncology, Unit of Endocrine, Digestive and Emergency Surgery, University Medical School of Bari "Aldo Moro", Bari, Italy; 2 Department of Emergency and Organ Transplantation, Unit of Endocrinology, University Medical School of Bari "Aldo Moro", Bari, Italy; 3 Department of Neurological and Psychiatric Sciences, Unit of Physical and Rehabilitation, University Medical School of Bari "Aldo Moro", Bari, Italy; 4 Unit of Otorhinolaryngology; "Di Venere Hospital", Bari, Italy

**Keywords:** Thyroid surgery, Emergency Surgery, Thyroid emergency, Hemorrhage, Acute Air Obstruction

## Abstract

**Background:**

Acute respiratory failure due to thyroid compression or invasion of the tracheal lumen is a surgical emergency requiring urgent management. The aim of this paper is to describe a series of six patients treated successfully in the emergency setting with total thyroidectomy due to ingravescent dyspnoea and asphyxia, as well as review related data reported in literature.

**Methods:**

During 2005-2010, of 919 patients treated by total thyroidectomy at our Academic Hospital, 6 (0.7%; 4 females and 2 men, mean age: 68.7 years, range 42-81 years) were treated in emergency. All the emergency operations were performed for life-threatening respiratory distress. The clinical picture at admission, clinical features, type of surgery, outcomes and complications are described. Mean duration of surgery was 146 minutes (range: 53-260).

**Results:**

In 3/6 (50%) a manubriotomy was necessary due to the extension of the mass into the upper mediastinum. In all cases total thyroidectomy was performed. In one case (16.7%) a parathyroid gland transplantation and in another one (16.7%) a tracheotomy was necessary due to a condition of tracheomalacia. Mean post-operative hospital stay was 6.5 days (range: 2-10 days). Histology revealed malignancy in 4/6 cases (66.7%), showing 3 primitive, and 1 secondary tumors. Morbidity consisted of 1 transient recurrent laryngeal palsy, 3 transient postoperative hypoparathyroidism, and 4 pleural effusions, treated by medical therapy in 3 and by drains in one. There was no mortality.

**Conclusion:**

On the basis of our experience and of literature review, we strongly advocate elective surgery for patients with thyroid disease at the first signs of tracheal compression. When an acute airway distress appears, an emergency life-threatening total thyroidectomy is recommended in a high-volume centre.

## Background

Acute respiratory failure due to thyroid compression or invasion of the tracheal lumen is a surgical emergency requiring urgent management.

Total thyroidectomy is a routine elective operation, but exceptionally it has to be performed on an emergency basis especially when it is life-threatening due to airway obstruction [1-5].

Laryngo-tracheal compression may be caused by giant or cervico-mediastinal goiter, acute intra-thyroidal hemorrhage, anaplastic carcinoma, lymphoma, and metastases from breast, lung, gastro-enteric and renal cancer [6-12]. Bilateral recurrent laryngeal nerve infiltration by anaplastic cancer, lymphoma, metastasis can also result in vocal cord palsy with worsening dyspnoea [13].

Hemorrhage in cysts and adenoma of thyroid gland is a common asymptomatic event [6];

On the contrary, massive hemorrhage, severe enough to result in acute airway distress is exceptional and more frequently secondary to neck trauma rather than a spontaneous complication of thyroid disease [14-16].

The aim of this paper is to describe a series of six patients treated successfully in the emergency setting with total thyroidectomy because of ingravescent dyspnoea and asphyxia, as well as review related data reported in literature.

## Methods

During 2005-2010, of 919 patients treated by total thyroidectomy at our Academic Hospital, 6 (0.7%; 4 females and 2 men, mean age: 68.7 years, range 42-81 years) were treated in emergency. All the emergency operations were performed for life-threatening respiratory distress, and by the same surgeon (M.T.) with high level of thyroid surgical skill. The clinical picture at admission, clinical features, type of surgery, outcomes and complications are described below. Mean duration of surgery was 146 minutes (range: 53-260).

### Case 1

**Figure 1:**
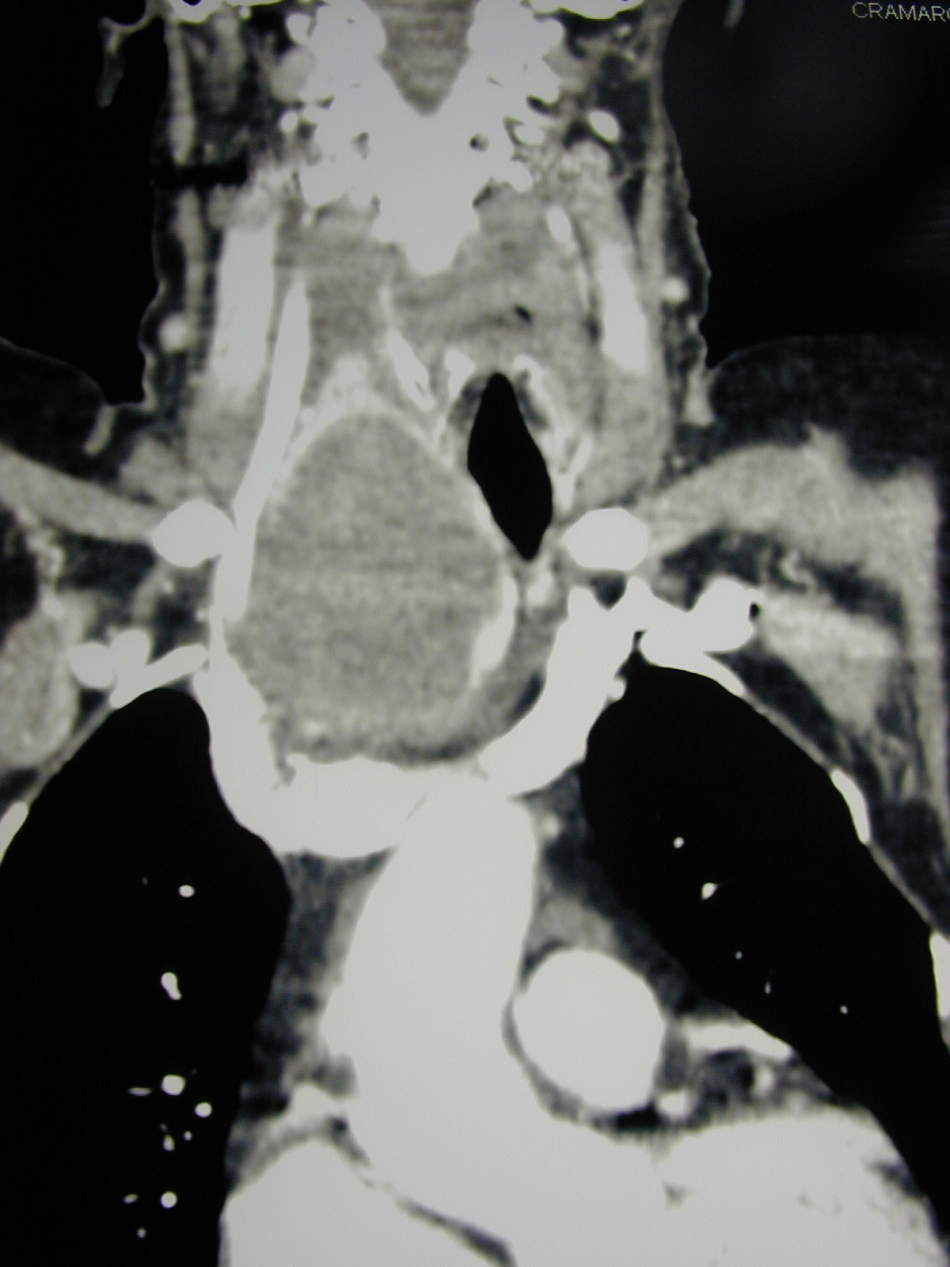
**Contrast enhanced CT scan, coronal reconstructed image**. The right lobe of the thyroid gland shows a voluminous mass compressing and dislocating trachea, and extending into the upper mediastinum.

**Figure 2:**
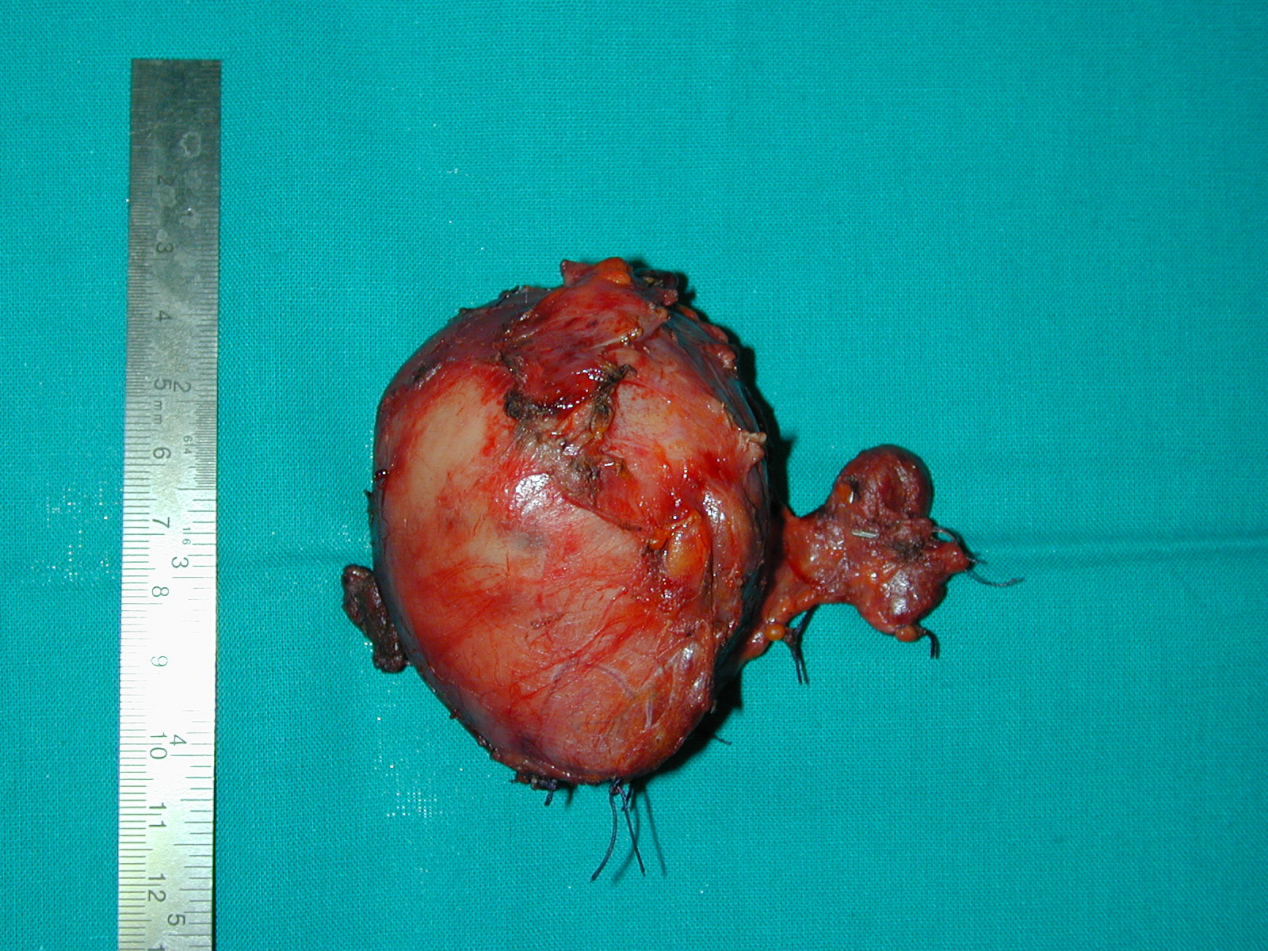
**Total thryroidectomy**.

An 81-year-old woman with dyspnoea, tachypnea, stridor, tachycardia, one week history of progressively increasing degree of breathlessness, and a 4-year history of anterior-lateral neck swelling came to our unit. Oxygen therapy was immediately set up, and an urgent CT scan of the neck (Figure 1) showed a huge multinodular goiter with retrosternal extension, producing left displacement of the trachea and its marked narrowing in laterolateral diameter. Because of rapidly worsening respiratory distress, an awake fiberoptic intubation using a small endotracheal tube, followed by induction of general anesthesia and emergency total thyroidectomy by manubriotomy were performed (Figure 2). Intraoperative surgical dissection helped by loupe magnification [17] revealed a mass adherent to the right common carotid artery and extending into the upper mediastinum. It also confirmed the marked left displacement of the trachea and permitted bilateral parathyroid gland and recurrent laryngeal nerve identification. Recovery showed a successfully treated atrial fibrillation and dysphonia due to a left vocal cord palsy confirmed by laryngoscopy. The patient was discharged 7 days after the operation. Microscopic examination revealed a Hürthle cell carcinoma. Transient recurrent laryngeal nerve palsy was successfully treated by logotherapy over a period of four months. The patient currently shows a five-year disease-free follow up.

### Case 2

**Figure 3:**
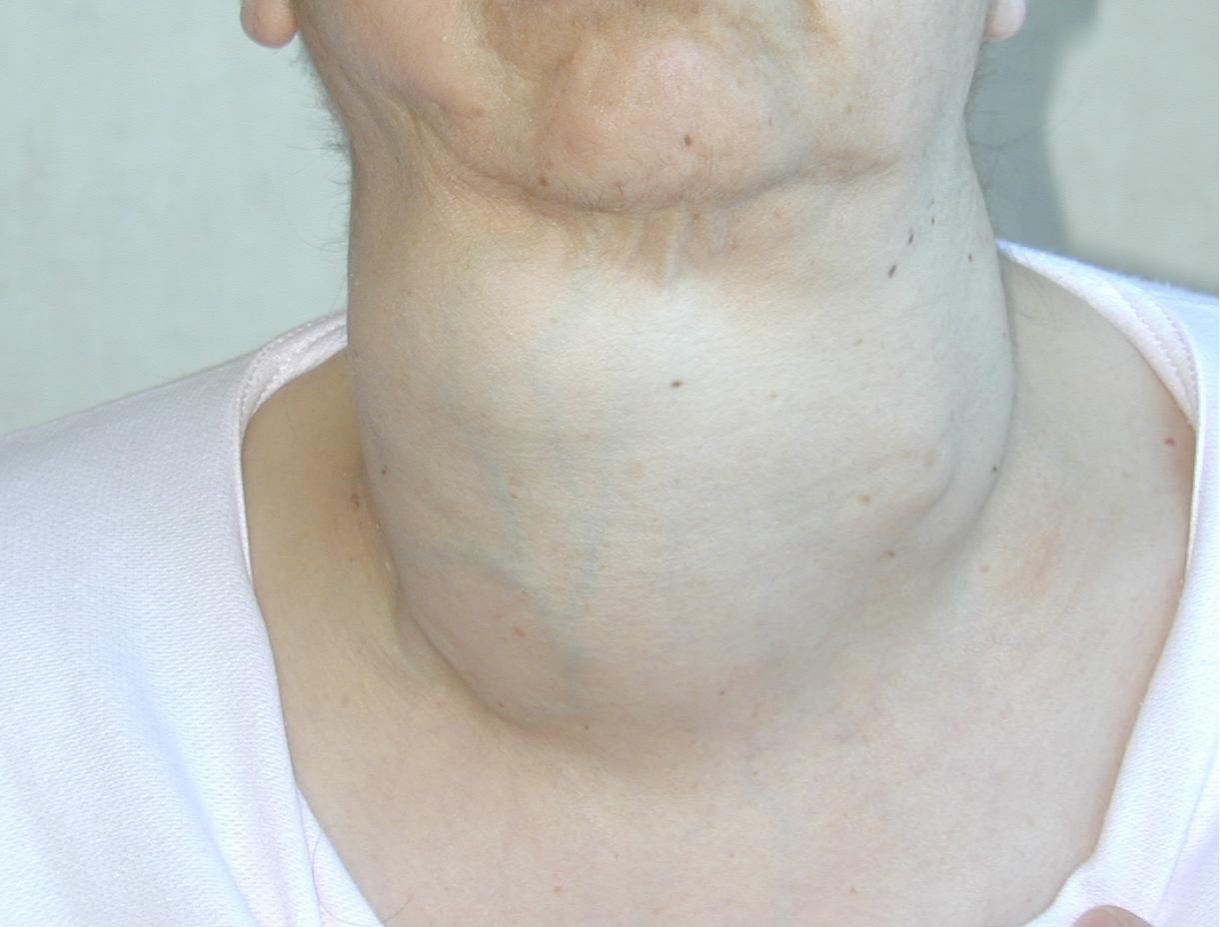
**Large and mainly right-sided cervical mass**.

**Figure 4:**
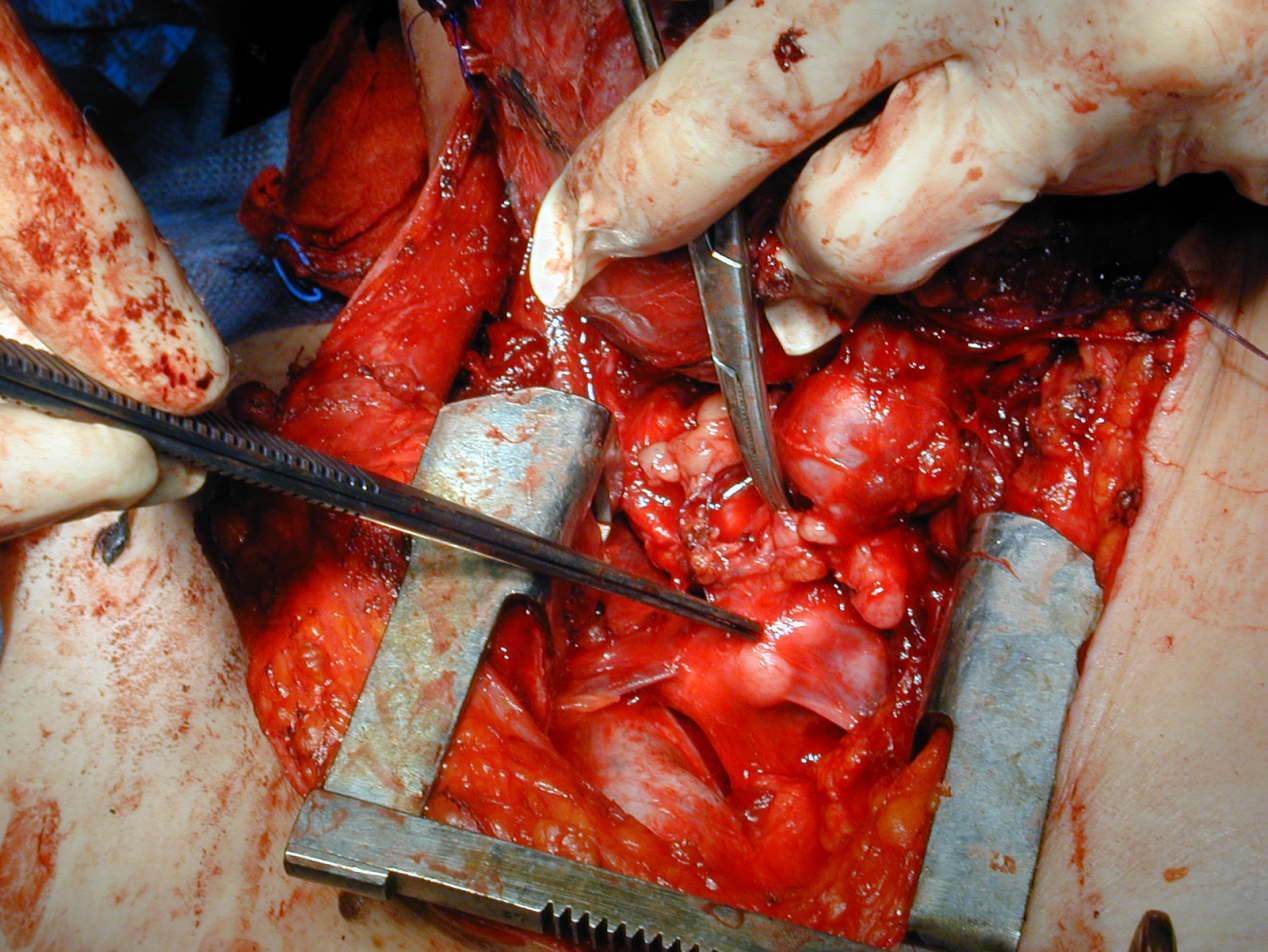
**At operation, performed by sternal split, the lumen of the trachea seemed to be almost completely shut by the compression of the mass**.

**Figure 5:**
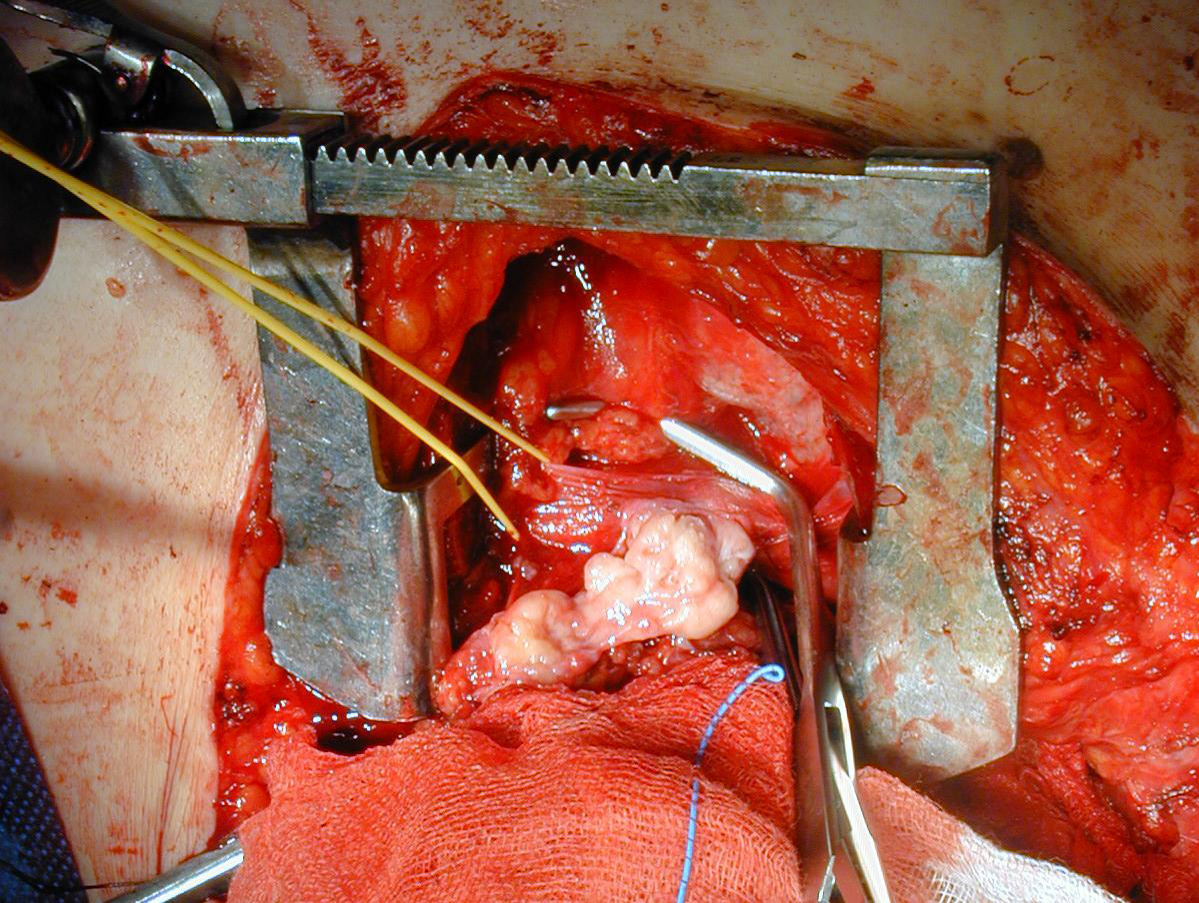
**The lower portion of this retrosternal goitre projected into the left innominate vein, with tumor floating into the lumen**.

**Figure 6:**
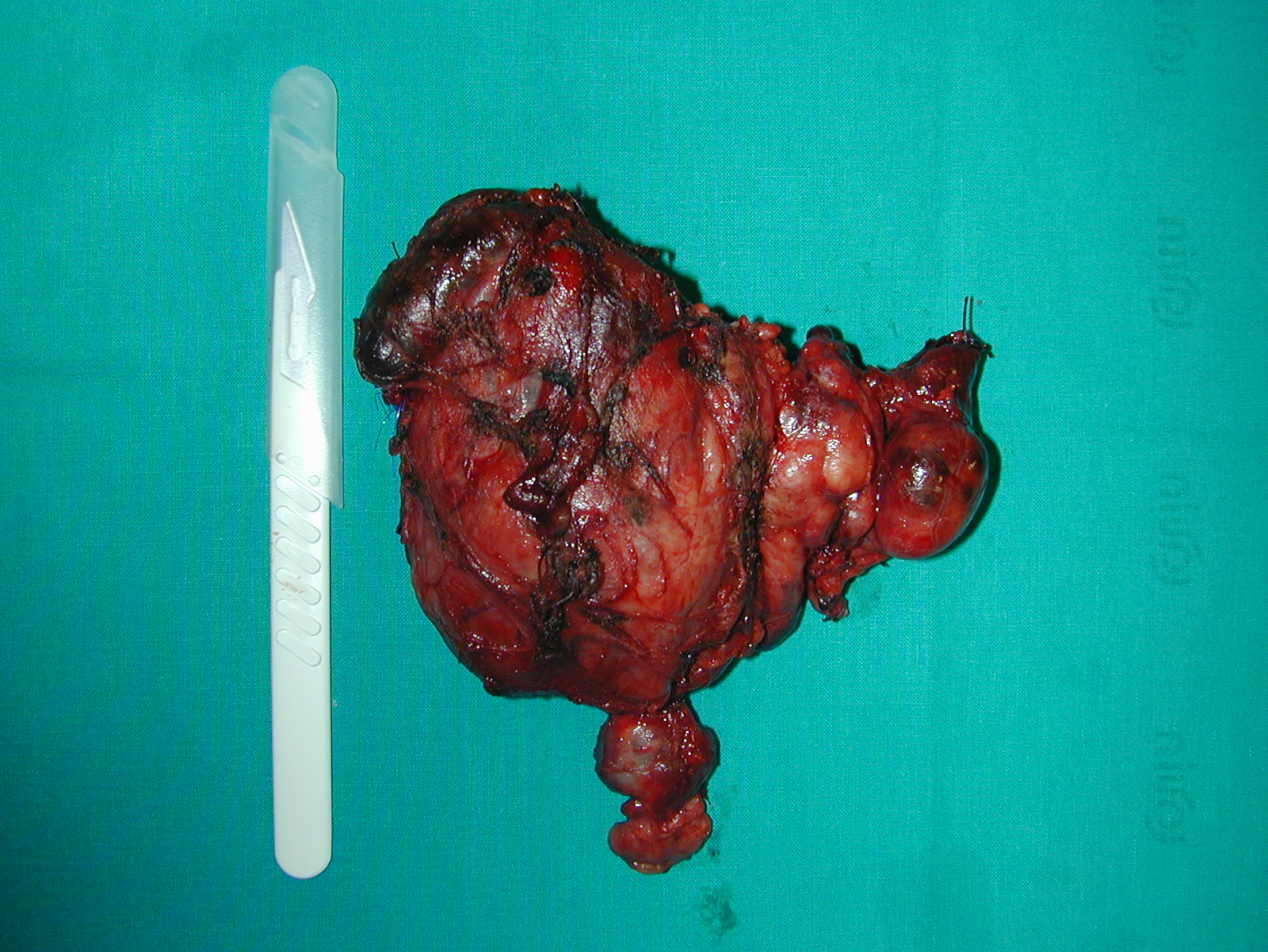
**Removal of the neoplastic thrombus through an incision in the vein was performed *en bloc *with the thyroid mass**.

A 59-years-old woman with a large and mainly right-sided cervical mass (Figure 3) came to us with severe dyspnoea, stridor and visible use of accessory respiratory muscles, and cyanosis. Computed tomography scan was performed after an awake fiberoptic intubation followed by induction of general anesthesia, revealing a thyroid mass extending into the upper mediastinum, with displacement and compression of the right jugular vein and carotid artery on the lateral side and of the trachea on the medial one, with an apparent adherence to the superior vena cava and left innominate vein. Emergency surgery was performed. At operation, performed by sternal split, the lumen of the trachea seemed to be almost completely shut by the compression of the mass, and the lower portion of this retrosternal goitre projected into the left innominate vein, with tumor floating into the lumen (Figures 4, 5). Removal of the neoplastic thrombus through an incision in the vein was performed *en bloc *with the thyroid mass (Figure 6). Both tumor and thrombus were completely replaced by follicular carcinoma. Recovery was uneventful and the patient was discharged ten days after the operation. After four years, and after radioiodine therapy and chemotherapy, the patient is still in follow-up without recurrence or evidence of metastases.

### Case 3

**Figure 7:**
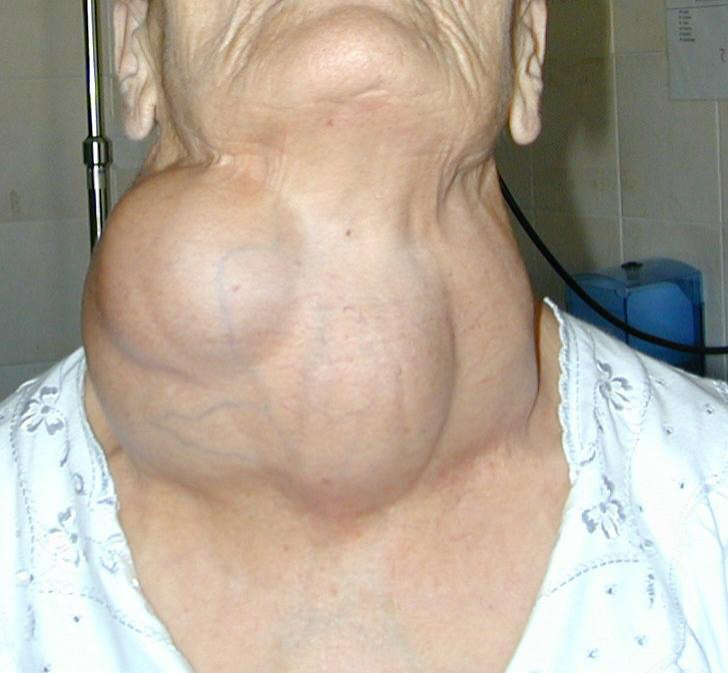
**Giant cervical goiter**.

**Figure 8:**
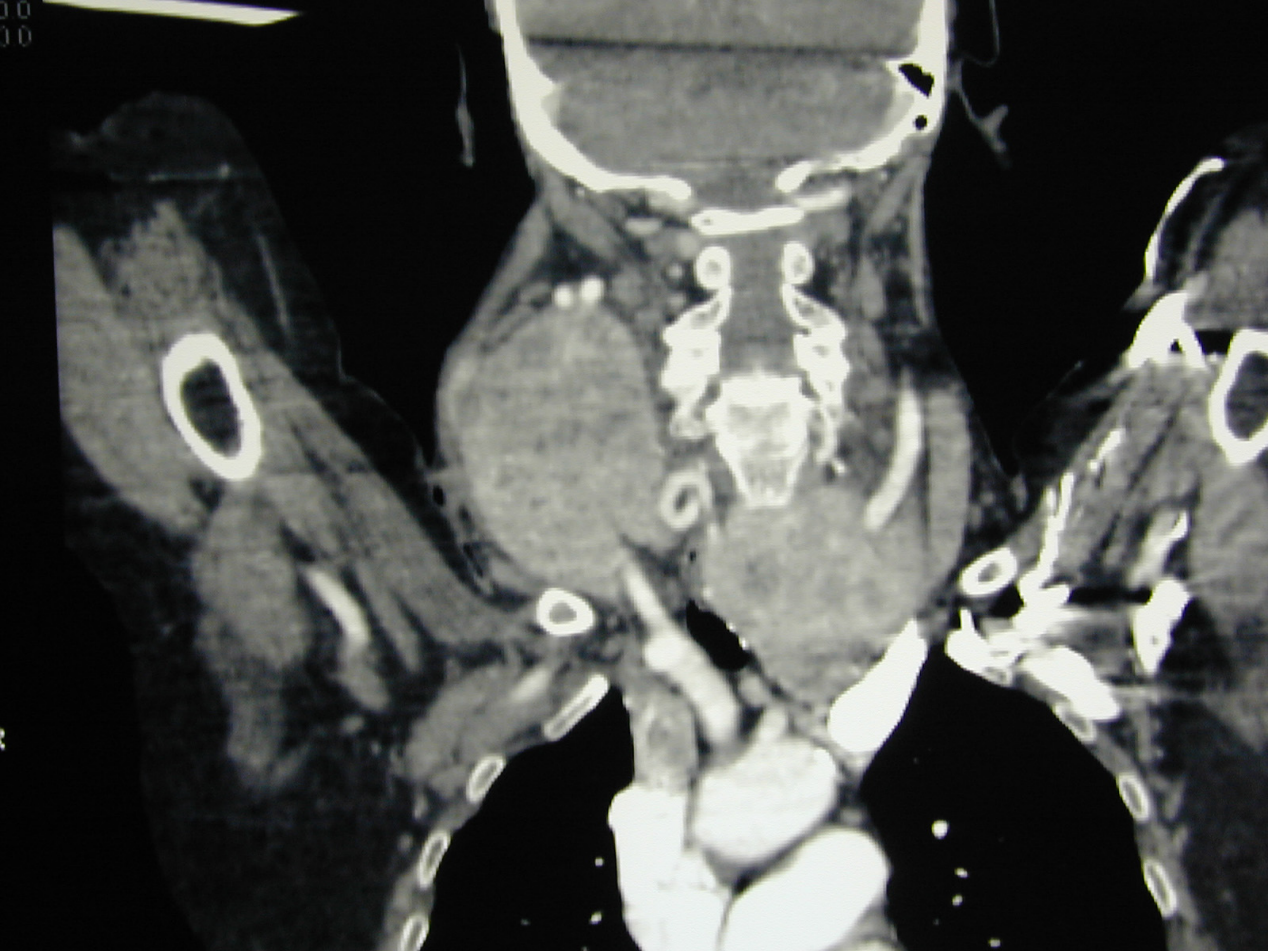
**Contrast enhanced CT scan, coronal reconstructed image**. A thyroid mass extending from the submandibular and submental regions to the parapharyngeal space and superior mediastinum is evident.

A 76-years old women was admitted in emergency with severe worsening respiratory distress due to a giant cervical goiter limiting cervical movements (Figure 7). Medical history revealed a developing mass over the past 50 years without toxic symptoms, increasing dysphagia and worsening ortopnea and paroximal dyspnoea. Physical examination revealed audible wheezing, inspiratory stridor, respiratory rate of 36 cycles/minute, with accessory respiratory muscles use, and tachycardia. Trachea was not reachable during palpation and carotid pulse was unpalpable on the right side and barely palpable on the left side. An urgent CT scan showed a thyroid mass extending from the submandibular and submental regions to the parapharyngeal space and superior mediastinum (Figure 8). A total thyroidectomy was performed in emergency under general anesthesia with a parathyroid gland autotrasplantation in the left sternocleidomastoid muscle according to our indications [18].

**Figure 9:**
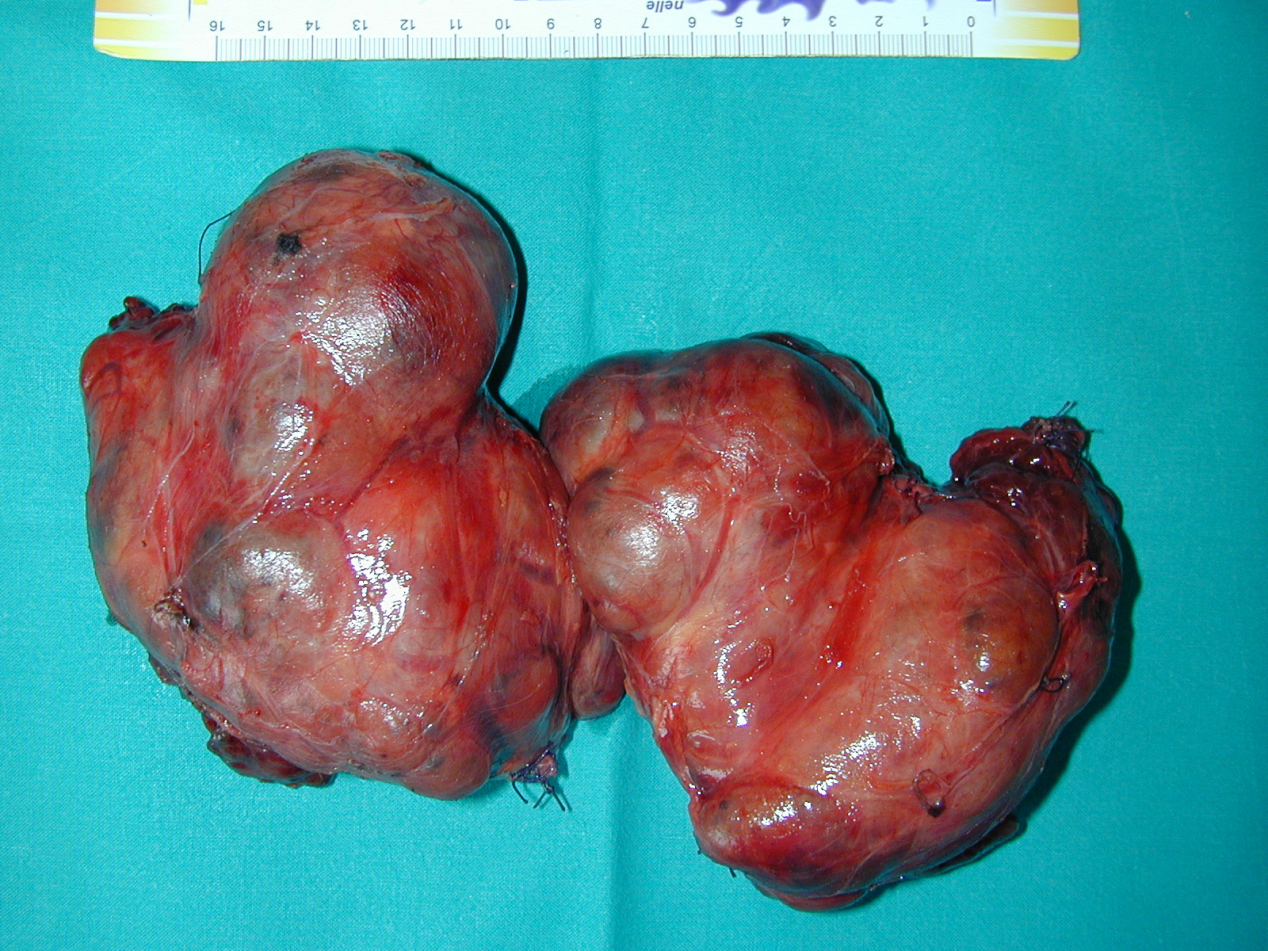
**thyroid gland measuring 23 × 16 × 12 cm and weighing 950 g**.

The recovery was uneventful and the patient was discharged on the third post-operative day. Pathologic examination revealed a thyroid gland measuring 23 × 16 × 12 cm and weighing 950 g (Figure 9), without histological signs of malignancy.

### Case 4[12]

**Figure 10:**
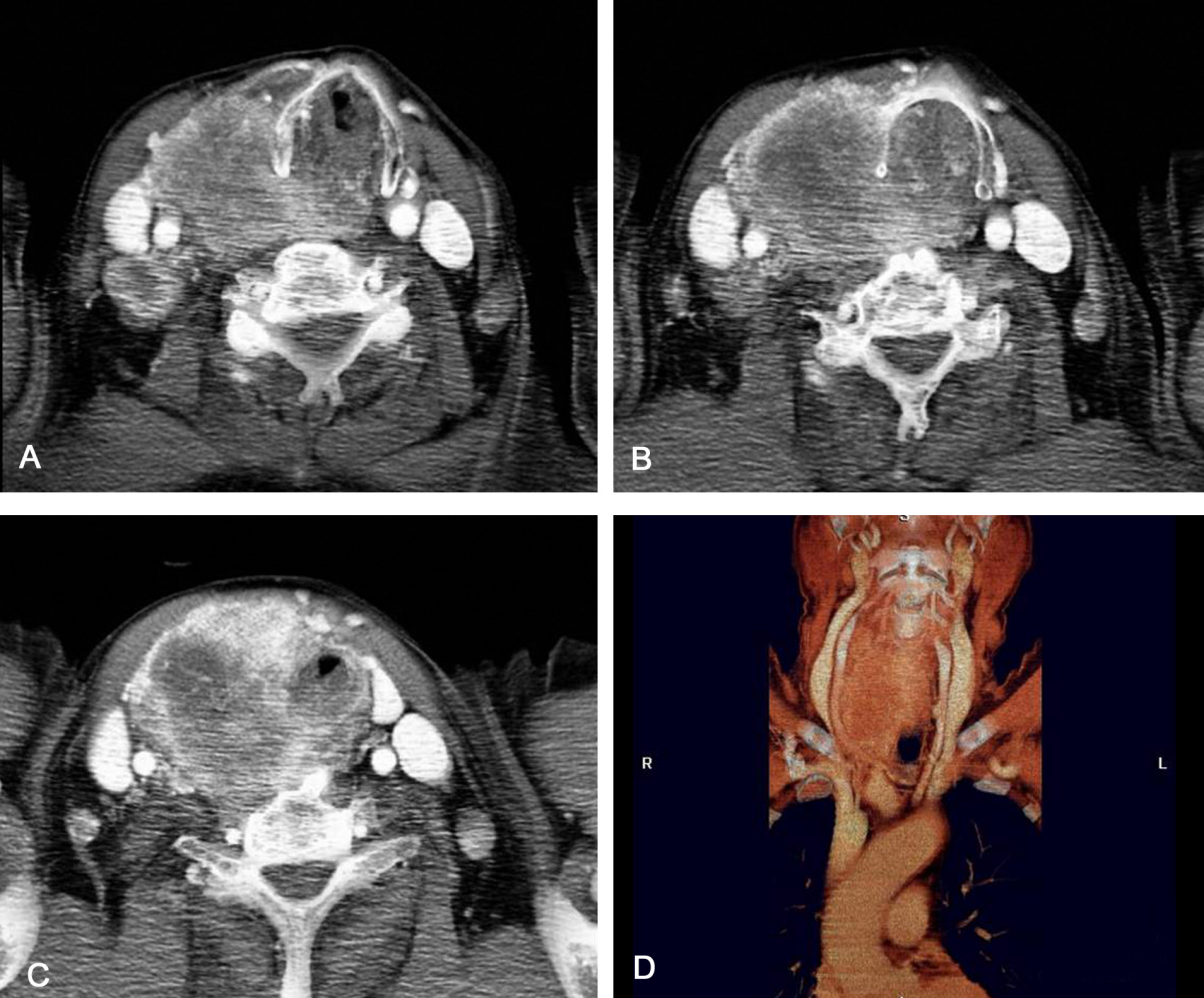
**Contrast enhanced CT scan, axial images and coronal reconstructed image**. Axial images sequences show the complete closure of the tracheal lumen. A thyroid mass with substernal extension, and with right jugular vein and carotid artery compression and displacement, in addition to diffuse lymphadenopathy are also evident.

**Figure 11:**
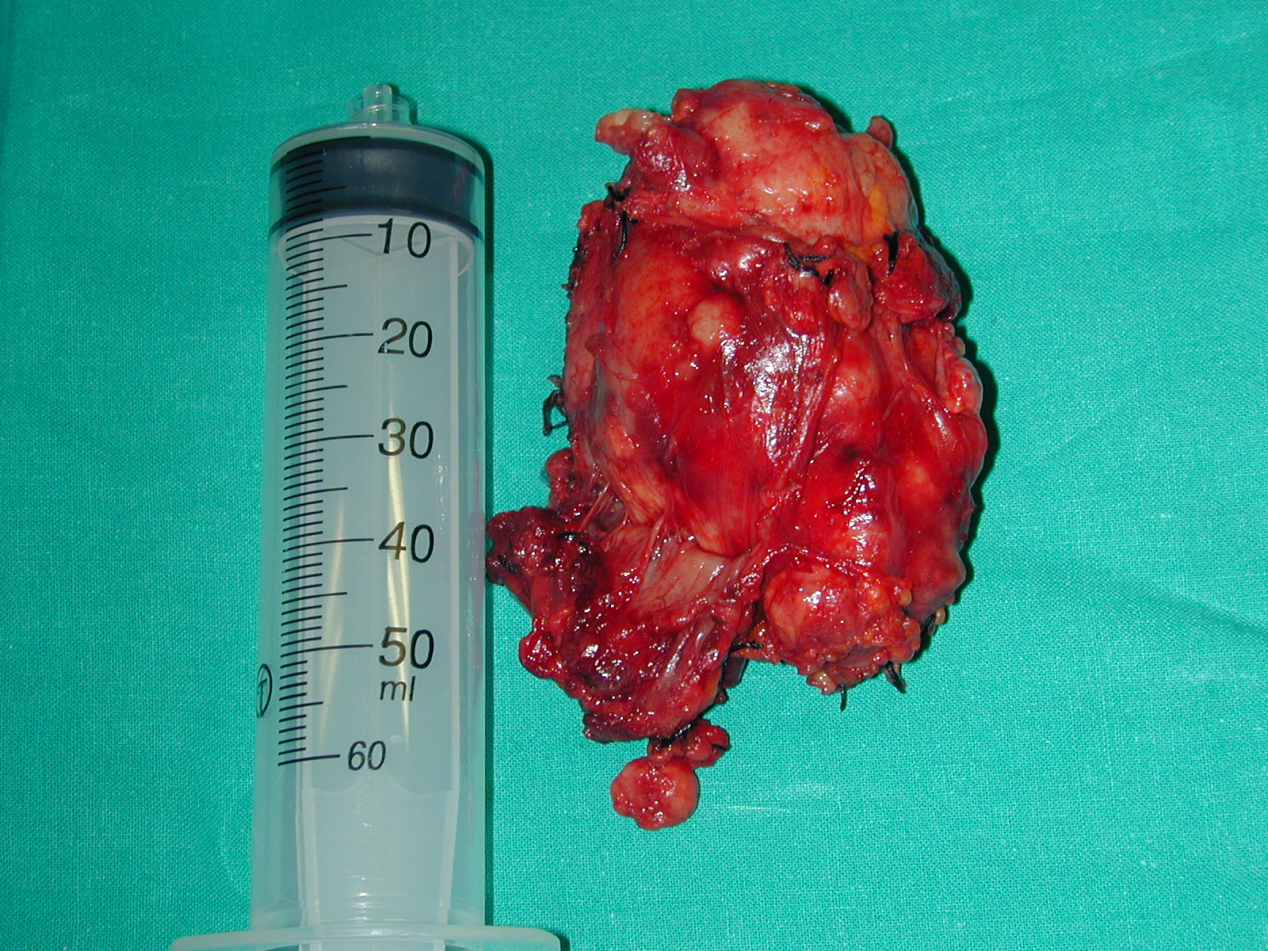
**Total thyroidectomy**.

**Figure 12:**
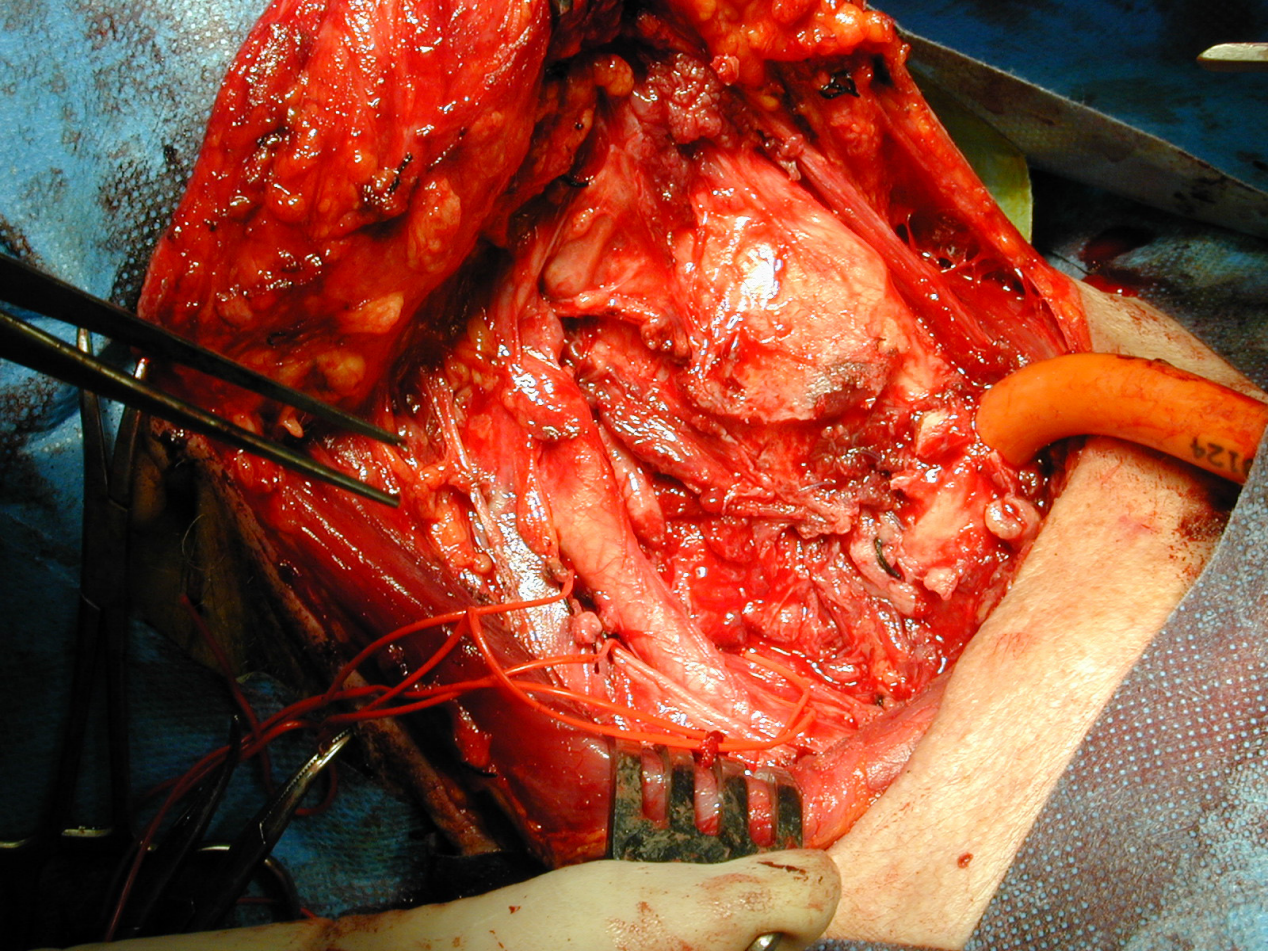
**Tracheostomy due to evident tracheomalacia**.

A 73-year-old man was admitted in emergency with a neck mass, sudden dyspnoea, stridor, dysphonia, and progressively worsening dysphagia. A history of multinodular goitre was noted in addition to a previous right radical nephrectomy for non-metastatic renal cell carcinoma 8 years before. The patient underwent fine-needle aspiration consistent with multinodular goitre 5 months before. Three days before admission the patient underwent a total-body CT scan showing a thyroid mass with substernal extension involving and completely obstructing the upper airways, the right vocal cord, with right jugular vein and carotid artery compression and displacement, in addition to diffuse lymphadenopathy (Figure 10). Physical examination revealed a large, painful, diffuse, and predominantly rightsided thyroid swelling. A flexible laryngoscopy revealed right vocal cord palsy and left cord paresis, with an almost total reduction of the laryngeal lumen. For these reasons, emergency endotracheal intubation was performed followed by total thyroidectomy with lymph node dissection (Figure 11). The operation was completed by a tracheotomy, considering the evident tracheomalacia (Figure 12). Histology revealed a poorly differentiated trabecular carcinoma, consisting of mainly clear cells with scanty oxyphil ones, with large nucleolated nuclei and frequent mitoses. Immunostains with alkaline phosphatase-anti-alkaline phosphatase showed strong and diffuse membrane positivity for CD10 antigen. These patterns were consistent with a renal cell primary carcinoma. The patient had an uneventful postoperative course and was discharged 10 days after the operation. Palliative chemotherapy was started, but the disease progressed and he died 7 months after surgery.

### Case 5[19]

**Figure 13:**
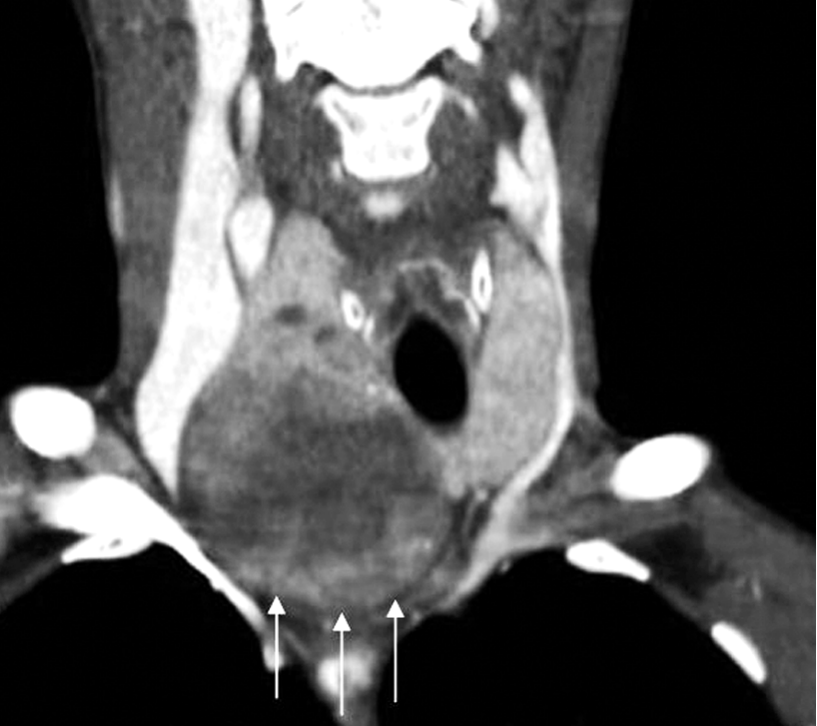
**Contrast enhanced CT scan with coronal reconstructed image: right lobe of the thyroid gland shows an inhomogeneous mass with focal areas of hemorrhage**. Compression and deviation of the trachea is also present.

**Figure 14:**
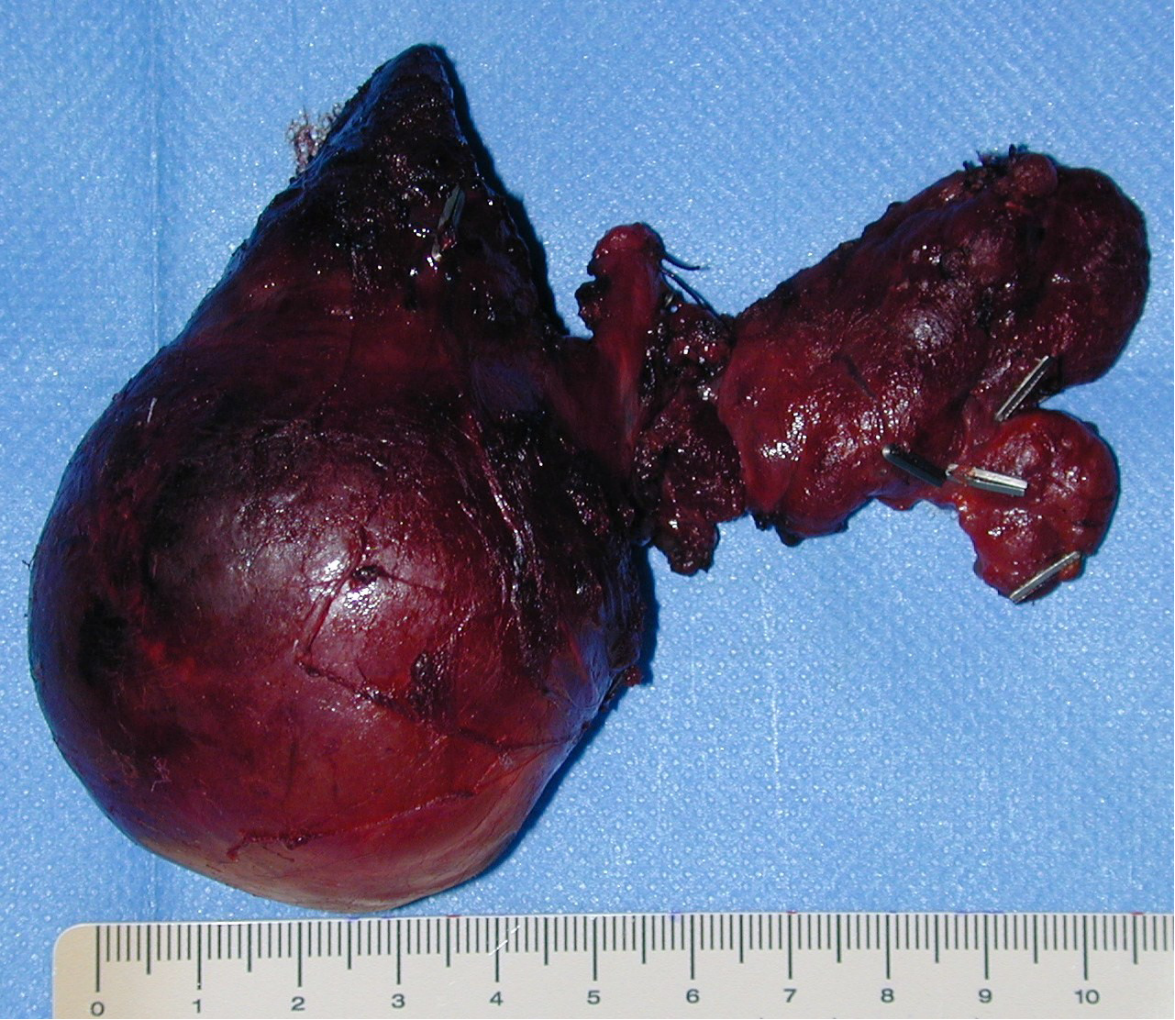
**Thyroid gland revealing a huge, edematous, nonfluctuant, rubbery, firm swelling with easy bleeding on touch, but the capsule appeared to be intact without rupture**.

A 42-year-old woman with a previous history of no symptomatic multinodular goiter was evaluated for an emergency at our surgical department for acute, progressive dyspnea and intermittent inspiratory stridor, associated with a spontaneous and rapidly enlarging mass of the neck. Clinical examination revealed a large, firm, nonfluctuant thyroid swelling on the right side of the neck. Initial analyses of arterial blood gas, complete blood cell count, electrolyte levels, prothrombin and bleeding times, and thyroid function tests were normal. An urgent computerized tomography scan showed a hematoma within the right lobe of the thyroid, with substernal extension, and tracheal deviation with marked luminal narrowing (Figure 13). The rapid progression of respiratory distress meant performing endotracheal intubation by flexible laryngoscopy revealing normal vocal cord function and an emergency total thyroidectomy. During the operation, the thyroid gland revealed a huge, edematous, nonfluctuant, rubbery, firm swelling with easy bleeding on touch, but the capsule appeared to be intact without rupture (Figure 14). Microscopic examination revealed a colloid multinodular goiter with massive parenchymal hemorrhage. Recovery was uneventful, and the patient was discharged 2 days after the operation.

### Case 6

**Figure 15:**
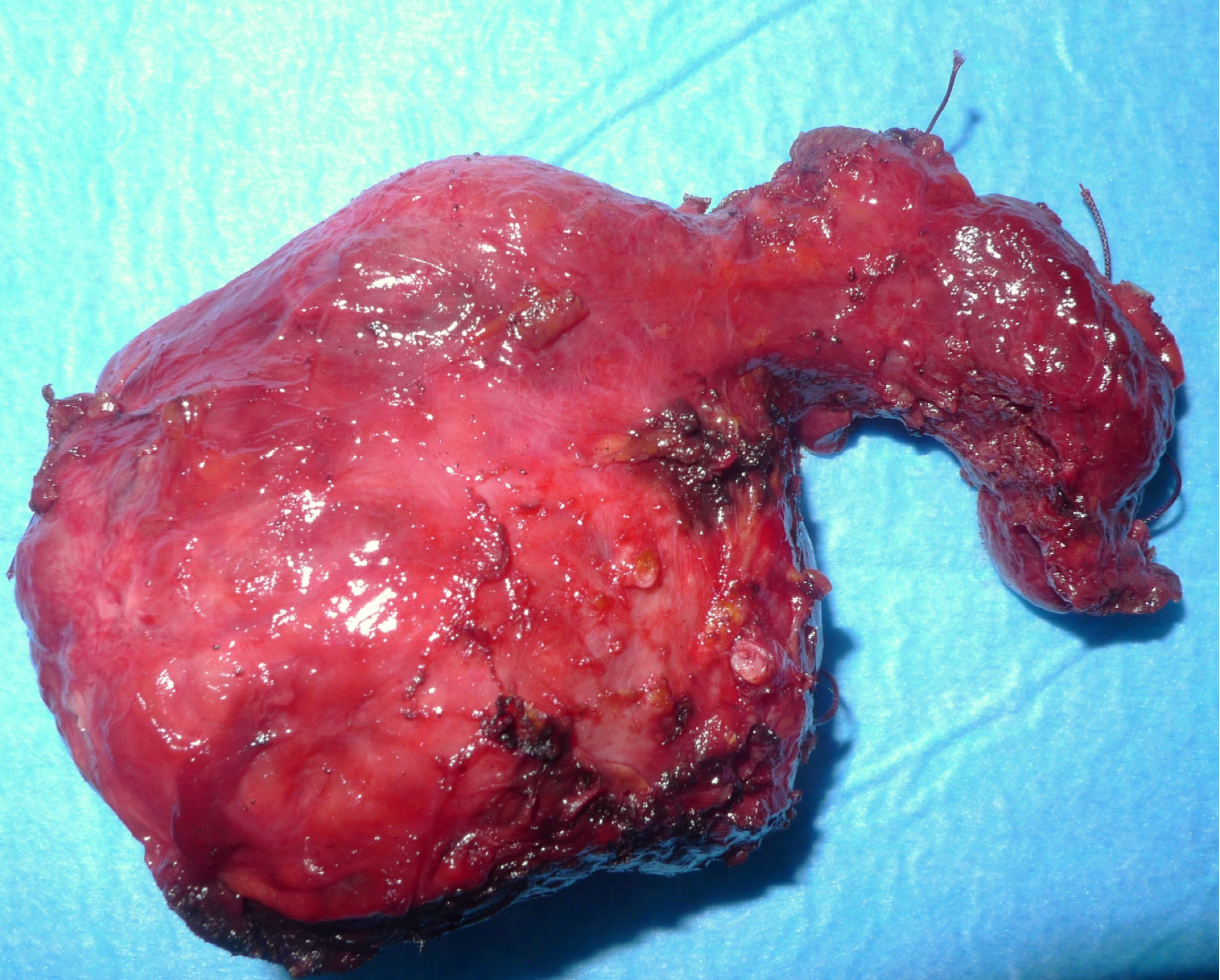
**Total thyroidectomy for a medullary carcinoma completely replacing the right lobe mass**.

An 81-year-old man with a forty-year history of substernal multinodular goiter was admitted in emergency with dysphonia and intermittent, sudden dyspnoea, and stridor. A flexible laryngoscopy revealed right vocal cord palsy, with a nearly total reduction of the laryngeal lumen, and a CT scan confirmed the compression of the trachea by a cervicomediastinal goitre. An emergency endotracheal intubation was performed followed by total thyroidectomy by manubriotomy. The thyroid gland appeared wooden in consistency, strongly adherent to the trachea, and to the pre-thyroid muscles, without signs of infiltrations, caudally extending up to the left *Innominate *vein (Figure 15). The patient was discharged seven days after the operation without postoperative complications. Histology revealed a medullary carcinoma completely replacing the right lobe mass. A follow-up of four months showed a normal calcitonin haematic level.

## Results

In 3/6 (50%) a manubriotomy was necessary due to the extension of the mass into the upper mediastinum. In all cases total thyroidectomy was performed by *3× *loupe magnification [17] to aid dissection of parathyroid glands, and recurrent laryngeal nerves. In one case (16.7%) a parathyroid gland transplantation [18] and in another one (16.7%) a tracheotomy was necessary due to a condition of tracheomalacia. Mean post-operative hospital stay was 6.5 days (range: 2-10 days). Histology revealed malignancy in 4/6 cases (66.7%), showing 3 primitive, and 1 secondary tumors. Morbidity consisted of 1 transient recurrent laryngeal palsy, 3 transient postoperative hypoparathyroidism, and in 4 pleural effusions, treated by medical therapy in 3 cases and by drains in one. There was no mortality.

## Discussion

In spite of *Hedenus *reporting successful thyroidectomies in six patients for goiters, which he described as "suffocating" [20] in 1821, nowadays airway obstruction due to goiter is exceptionally reported in literature [2-5,7,9,14] due to improved diagnostic methods and earlier treatment.

Although this dramatic occurrence seems to be more frequent in developing countries due to ignorance and lack of ready access to affordable medical services, in western countries the phenomenon of giant goiters is very uncommon though not completely absent [21,22]. A truly severe life-treating airway obstruction is, therefore, currently an extremely rare event [2,21,23,24], also because the tracheal lumen may be progressively compressed without causing symptoms up to 75% [2].

The causes of severe respiratory distress related to non traumatic thyroid disease show four different etiopathogeneses: rapidly progressive pressure on the tracheal lumen by spontaneous intrathyroideal hemorrhage, invasion of the tracheal lumen by primitive or secondary tumors, severe compression from benign or malignant masses and bilateral vocal cords palsy resulting from infiltration of recurrent nerves from thyroid malignancy.

Among the causes, spontaneous hemorrhage is often but not always [25] related to benign condition and is paradoxically the most insidious because it suddenly and unexpectedly appears in its full strength, sometimes in patients without previous history of thyroid disease; consequently diagnosis may be delayed. Indeed, literature [26-28] reports mortality related to this event of up to 27.8% [26]. The most likely explanation for hemorrhage in goiters is thought to be venous bleeding [19]. The adenomatous goiters are usually more fragile than normal thyroid because of the increased vascular flow and the lack of a true capsule; these aspects easily explain the great propensity for injury by blunt trauma [29], or iatrogenic bleeding resulting from fine-needle aspiration biopsy [30,31].

In the spontaneous thyroid hemorrhage, however, the mechanism is unclear. Johnson [32] and Terry [33] proposed that the inciting event for the hemorrhage was increased venous pressure resulting from the *Valsalva *maneuver. Therefore, most spontaneous cases are found to have an associated external event, such as various forms of light housework, coughing, straining at defecation, crying, which are, however, seemingly insignificant [6]. However, on appearance of a stable spontaneous hematoma of the thyroid gland without airway compromise, we believe that a conservative treatment could be safely performed; however, the increasing size of neck swelling or acute worsening of respiratory distress must be absolute, although rare indications point to early intubation and emergency surgery [19], as in the case reported in this series.

Anaplastic thyroid cancer is a rare tumor, ranging from 1-3% of all thyroid neoplasms, but is characterized by a very aggressive loco-regional disease, with mortality often related to respiratory failure from infiltration of the tracheal lumen [34]. Indeed, the main indication for surgery is just palliative decompression and debulking to prevent invasion of larynx, trachea, nerves and vessels of the neck, in the presence of a median survival of 4-5 months from the time of diagnosis [25].

Thyroid lymphoma [35], and leiomyosarcoma [36] are exceptionally described as causes of tracheal obstruction with respiratory distress treated by total or partial thyroidectomy.

On the other hand, well-differentiated thyroid carcinoma may, on occasion, cause airway obstruction [37]. The usual treatment of carcinoma invading the trachea is by "shaving" the tumor off the trachea, expecting to control residual neoplasm by postoperative radioactive iodine or external irradiations therapies [37,38]. However, the prognosis for well-differentiated carcinomas worsens when the neoplasm invades the trachea; indeed, the cause of death in nearly half of the fatal cases of papillary carcinomas is caused by obstruction of the trachea [37,39]. Moreover, the survival rate of patients treated by incomplete resection of the affected trachea is much worse than patients treated by complete resection [40,41]. For these reasons, with progress in tracheal surgical techniques, resection of portions of the trachea with primary anastomosis *en bloc *with thyroid is nowadays the treatment of choice [40-43]. Four cases (66.7%) in this reported series were well-differentiated carcinoma. In case 1, 2, and 6 (Hürthle cell, follicular, and medullary carcinomas, respectively), the airway obstruction was determined by the compression but not by the infiltration of trachea from the thyroid mass, and a comfortable cleavage plain between trachea and thyroid was evident at operation during dissection. For this reason a trachea resection was deemed unnecessary and the long-term disease-free follow up provides proof of the correctness of the surgical decision. In case 4 (thyroid metastasis from renal cancer), however, despite the invasion of the trachea, the staging of a metastatic disease contraindicated resection. Indeed, the patient died 7 months after the operation, due to the disease progress, but without local recurrence.

When the respiratory distress is caused by benign thyroid disease, usually the compression *ab estrinseco *of the trachea is determined by a giant cervical or cervicomediastinal goiters. However, there being enough room to accommodate the gland, acute respiratory failure secondary to tracheal compression by goiter is extremely rare [2], affecting 0.6% of reported cases [44]. However, when the extension of the goiter is retroclavicular, it can cause airway obstruction that may progress to arrest respiration [2,45,46]. Nevertheless, in the presence of benign thyroid disease, chronic obstructive airways disease, substernal extension, and long-standing goiter are considered as risk factors for developing acute, life-threatening airway compromission [44].

It is clear that the appearance of an acute airway obstruction requires urgent management to ensure an adequate ventilation and oxygenation.

The first step in the management of this emergency is represented by the anesthesia. An awake fiberoptic intubation using a small endotracheal tube followed by induction of general anesthesia, as always performed in this reported series, seem to be the gold standard in the approach to this emergency. Indeed, a standard sequence of induction and intubation could be considered at risk of aspiration in an unfasted patient, and besides this, the possibility of unsuccessful intubation due to the compression by the goiter is very high. On the other hand, an inhalation induction followed by laringoscopy and orotracheal or blind nasal intubations, may be considered dangerous because of complete airway obstruction following loss of consciousness [47,48]. When assisted intubation cannot be achieved, local or regional anesthesia are described too [21].

The second step is the choice of surgical treatment to be performed. Indeed, surgery - emergency or early - is always indicated for severe airway obstruction caused by thyroid mass [23]. An emergency tracheostomy is hindered by the presence of the thyroid mass which prevents access to the trachea, obliterating all landmarks [21]. An isthmectomy to allow a tracheostomy, appears to be an incomplete treatment, referring to a second surgical procedure for removing the entire thyroid. Moreover, in the presence of diagnosis of proven or suspected malignancy, it would cause a further delay in cancer treatment and exposes the patient to the risk of tumor dissemination. However, even in the presence of a benign goiter, re-surgery would mean higher morbidity [49,50]. Finally, once an endotracheal intubation has been performed, tracheotomy is questionable. Since a total thyroidectomy is capable of resolving airway obstruction, tracheostomy would result in unnecessary discomfort for the patient, furthermore exposing then to the need of a second operation to close the stomy. In our experience tracheostomy was necessary in only one case (16.7%) due to the evidence of a marked tracheomalacia.

Then, total, near-total or sub-total thyroidectomy represents the treatment of choice of acute airway obstruction resulting from compression of thyroid mass.

On the basis of our experience and that in literature, in the presence of warning signs such as a developing mass in patients with history of thyroid disease, increasing and/or intermittent dyspnoea, stridor, sudden onset of neck swelling, we stress the importance of immediate hospitalization to perform a controlled approach to the progressively acute disease, avoiding treatment in emergency. But, when this event occurs, like in our reported series, the approach to this emergency operation should be performed in highly specialized high-volume centers combining multidisciplinary anesthesiological and surgical strategies. Indeed, when total thyroidectomy is performed for cervicomediastinal goiters, there is a higher risk of postoperative hypoparathyroidism, recurrent laryngeal nerve palsy and hemorrhage, as reported in literature [8,51-57] and in our experience too, [58] which sometimes requires sternal split, as in 50% of this series. However, in our experience, the use of loupe magnification and parathyroid autotransplantation during thyroid surgery showed a significant improvement of results, with faster and safer identification of the nerve, and decreasing permanent and transient hypoparathyroidism [17,18]. Some authors suggest the use of the recurrent nerve monitor, especially in the presence of a large retrosternal goiter [59,60].

Moreover, when the upper mediastinum is occupied by a goiter, the endocrine surgeon is not usually familiar with the course of the RNLs and their anatomical variability in this district, and the cardiothoracic surgeon is not familiar with endocrinosurgical challenges. Therefore, the emergency extracervical approach could require multidisciplinary collaboration [58].

In conclusion, on the basis of our experience and of the literature review, we strongly advocate elective surgery for patients with thyroid disease at the first signs of tracheal compression. When an acute airway distress appears, an emergency life-threatening total thyroidectomy is recommended in a high-volume centre.
